# Variations in olfactory function among bipolar disorder patients with different episodes and subtypes

**DOI:** 10.3389/fpsyt.2023.1080622

**Published:** 2023-03-20

**Authors:** Chunyang Li, Liu Hong, Laiquan Zou, Yiping Zhu, Jianfu Ye, Fenlan Wu, Chao Chen

**Affiliations:** ^1^Department of Psychiatry, Shunde Wu Zhongpei Hospital, Foshan, Guangdong, China; ^2^Department of Psychology, School of Public Health, Southern Medical University, Guangzhou, China

**Keywords:** bipolar disorder, olfactory function, olfactory sensitivity, olfactory identification, Sniffin’ Sticks test

## Abstract

**Purpose:**

Most studies on olfactory function in individuals with bipolar disorder (BD) have not distinguished between the different subtypes or between the acute phase (mania or depression) and euthymic state. In this study, we compared olfactory function among BD patients with different subtypes and episodes to explore the potential use of olfactory function as a biomarker for the early identification of BD.

**Patients and methods:**

The study sample consisted of 117 BD patients who were hospitalized between April 2019 and June 2019, and 47 healthy volunteers as controls. The BD patients were divided into a bipolar I disorder (BD I) (*n* = 86) and bipolar II disorder (BD II) group (*n* = 31) according to the different subtypes, and divided into depressive BD (*n* = 36), manic BD (*n* = 44), or euthymic BD (*n* = 37) groups according to the types of episodes they experienced. We assessed olfactory sensitivity (OS) and olfactory identification (OI) *via* the Sniffin’ Sticks test and used the Hamilton Depression Rating Scale (HAMD) and Young Manic Rating Scale (YMRS) to evaluate BD characteristics among all subjects.

**Results:**

Compared with controls, the participants with BD showed decreased OS and OI. We found statistically significant differences in OS and OI between the BD I group and controls, as well as differences in OS between the BD I and BD II group. Least-significant difference multiple comparisons revealed statistically significant differences in OS between the depressive BD group, manic BD group and controls and also between the manic BD and euthymic BD group. OI was positively correlated with the YMRS score in the BD I group and OS was negatively correlated with the HAMD score in the BD II group.

**Conclusion:**

This may be the first study to compare olfactory function in patients with BD I vs. BD II *via* pairwise comparisons. Our findings suggest that OS may have potential as a biomarker for distinguishing the different subtypes of BD and as a state-related biomarker for differentiating the acute phase from the euthymic state of BD. However, further prospective research is warranted.

## 1. Introduction

Bipolar disorder (BD) is a disabling disease characterized by severe emotional instability, accompanied by cognitive and functional impairment (1). Individuals with BD experience recurring manic or hypomanic episodes that sometimes alternate with depressive episodes. Bipolar I disorder (BD I) is characterized by apparent manic episodes and may result in distinct impairment of psychosocial function. Bipolar II disorder (BD II) is defined mainly by episodes of depression alternating with hypomania rather than mania (2).

An early correct BD diagnosis can contribute to an improved prognosis (3). However, the diagnosis of BD remains, to a great degree, a subjective clinical exercise. The development and validation of biomarkers for BD may be conducive to earlier diagnosis and a better treatment response, which are targets of precision psychiatry (4, 5).

Numerous studies have demonstrated a close relationship between olfaction and emotional information processing (6). As a result of the partial overlap between the brain regions involved in olfactory processing and those involved in the pathophysiology of psychiatric diseases, such as the limbic system and prefrontal structures, olfactory deficits are common (often prodromal) in individuals with neurodegenerative or psychiatric disorders (7–9). Therefore, changes in olfactory function have great potential as early biomarkers of disease (10). At present, psychological conditions such as schizophrenia (11, 12), depression (13, 14), anxiety disorder (15, 16), post-traumatic stress disorder (17, 18) and obsessive-compulsive disorder (19, 20), have been associated with olfactory defects.

Although several studies have examined olfactory function in BD patients, the results have been inconsistent. Some studies have shown abnormal olfactory function in BD patients (21, 22), while others have shown no significant differences compared with control groups (23–25). Specifically, some studies have revealed olfactory identification (OI) defects in BD patients (21, 22, 26, 27) while others show no OI defects in this population (23–25, 28–30). All studies showed normal olfactory sensitivity (OS) performance, with no significant differences compared with control groups (23, 24, 26, 28, 29), except for two studies. One of these found decreased OS in BD patients with acute phase BD (manic phase and depressive phase) (27) while the other reported increased OS in euthymic BD patients with event-related episodes compared with euthymic BD patients without such episodes (31).

Advances in imaging technology have illuminated the relationship between olfaction and BD. Takahashi et al. used MRI to find that BD I patients had a significantly shallower bilateral olfactory sulcus compared with controls, suggesting that neurodevelopmental abnormalities might function as static markers of BD (32). They also found that patients taking valproate had a longer bilateral sulcus compared with those who were not undergoing valproate treatment. Negoias et al. reported that patients with euthymic BD showed a stronger central responsiveness to olfactory stimuli during fMRI regardless of normal olfactory results, indicating that an over-activated brain network is part of olfactory or emotion processing circuits in BD patients (29).

Few previous studies on olfactory function in BD have distinguished between the different subtypes, or between the different episodes (manic or depressive) and the remission period. Kamath et al. (26) examined olfactory function in five groups (including BD I, BD II, major depressive disorder, anxiety, and controls) and found that OI was lower in BD I patients (only among those with psychotic features) compared with controls. However, they did not perform pairwise comparative analyses between the BD I and BD II groups. To address this, we conducted a cross-sectional study to compare olfactory function in BD patients with different subtypes or episodes. We hoped to determine whether olfactory function has potential as a biomarker for the early identification and differential diagnosis of BD. We hypothesized that OS and OI deficits would present in the BD I and BD II groups, with more severe symptoms in the former. The secondary objective was to compare olfactory function between manic or depressive and euthymic BD patients, and we hypothesized that olfactory dysfunctions would be worse in manic or depressive patients than in euthymic patients, which may serve as potential markers of the state of BD in patients.

## 2. Materials and methods

### 2.1. Participants

The study sample consisted of 117 BD patients who were hospitalized between April 2019 and June 2019, as well as 47 healthy volunteers as controls.

The inclusion criteria for BD patients were as follows: (1) diagnosis of BD according to the diagnostic criteria of the Diagnostic and Statistical Manual of Mental Disorders, Fifth Edition (DSM-5); (2) age 18–60 years; (3) Han nationality; (4) primary school education or above; and (5) the patient volunteered to participate in this study and signed the informed consent form.

The inclusion criteria for BD patients in the euthymic state were as follows. In addition to meeting the above inclusion criteria for BD patients, the subjects also met the following requirements simultaneously: (1) stable current condition, without obvious clinical symptoms; (2) Hamilton Depression Rating Scale (HAMD) score ≤7; and (3) Young Mania Rating Scale (YMRS) score ≤7.

The inclusion criteria for the healthy control group were as follows: (1) healthy adults without a history of mental illness; (2) HAMD score ≤7; (3) YMRS score ≤7; (4) age 18–60 years; (5) Han nationality; (6) primary school education or above; and (7) the individuals volunteered to participate in this study and signed the informed consent form.

The study exclusion criteria were as follows: (1) any physical diseases that may affect olfactory function, such as nasal polyps, chronic sinusitis, and other nasal or paranasal sinus diseases or surgery, acute upper respiratory tract infection in the past 2 weeks; (2) neuropsychiatric diseases that may affect olfactory function, such as Alzheimer’s disease, Parkinson’s disease, epilepsy, multiple sclerosis, and stroke; (3) moderate or severe cognitive impairment as revealed by Mini-Mental State Examination (MMSE) scores <20 (primary school or below) or <24 (junior high school and above); (4) a history of alcohol or drug abuse or dependence in the past year; and (5) inability to complete the olfactory function test.

All patients or their legal representatives and members of the healthy control group signed informed consent forms before beginning the study. The study followed the principle of voluntary participation and participants were able to withdraw from the study at any time. The study was approved by the Ethics Committee of Shunde Wu Zhongpei Hospital, Foshan City before the experiments began.

### 2.2. Study assessments

#### 2.2.1. Demographic and clinical data collection

We designed a questionnaire to collect basic demographic information including participant age, gender, place of origin, ethnicity, education level, smoking status, and physical health status. Similarly, we designed a clinical data registration form to record the participant diagnosis, clinical classification, episode type, course of disease, medication, psychotic symptoms, family history, and other clinical data for the BD group.

#### 2.2.2. Assessment tools

##### 2.2.2.1. HAMD

The 24-item version of the scale (HAMD-24) is used to assess the severity of depressive symptoms. A HAMD-24 score greater than 35 may indicate major depressive symptoms, a score greater than 20 may indicate mild or moderate depressive symptoms, and a score less than 8 may indicate no depressive symptoms (33). The assessment period generally includes the previous 2 weeks.

##### 2.2.2.2. YMRS

There are 11 items in total, most of which are graded from 0 to 4, and items 5, 6, 8, and 9 are graded from 0 to 8 to show the severity of disease in uncooperative patients. The YMRS total score reflects the symptoms and severity of mania. A score of 0–7 indicates no obvious symptoms of mania, while 8–12 indicates mild, 13–19 indicates moderate, 20–29 indicates severe, and a score of more than 30 indicates very severe symptoms (34). The assessment period is the previous week.

##### 2.2.2.3. Severity of illness rating scale

The severity of illness was assessed using the Clinical General Impression Scale-Severity of Illness (CGI-SI). Scores range from 0 to 7 points, where 0 indicates no disease, a score of 1 indicates basically no disease, and a score of 7 reflects very serious disease.

##### 2.2.2.4. Global assessment function (GAF)

Clinicians use this scale to comprehensively assess the psychological, social, and occupational functioning of subjects. The GAF scale ranges from 1 to 100. A lower score indicates a more serious impairment in social function.

##### 2.2.2.5. Sniffin’ Sticks test (SST)

A quantitative olfactory evaluation tool developed by Kobal and Hummel. In this study, we used the SST to evaluate OS and OI, and OS was assessed according to olfactory threshold. The OS test consisted of 48 olfactory sticks; with 16 sets containing 3 sticks each. The highest concentration of n-butanol, which was the olfactory agent on the sticks, was 4%. The agent was diluted in 1–16 grades, and higher scores were given for the correct identification of sticks with lower concentrations. Higher OS scores indicated better olfactory sensitivity. The OI test consisted of 16 olfactory rods, with fragrances such as oranges, leather, chocolate, and mint. Higher scores on the OI test indicated better OI (35). OS is generally considered to reflect functioning of the peripheral olfactory pathway, particularly related to the occurrence of olfactory bulb cells and the regeneration of olfactory epithelial cells. OI is an indicator of central olfactory function, which requires the participation of higher cognitive functions, especially memory, attention and executive functions, and thus involves numerous brain regions, including the hippocampus, amygdala and anterior cingulate gyrus.

The scales and olfactory test were carried out in a quiet, ventilated, and odor-free environment, which took about 60 to 80 min to complete for every participant. Two psychiatrists trained in the use of the above scales acted as evaluators, and participated in the assessment of each subject. After each assessment, the scores for each scale were discussed, and the consensus score was generated.

### 2.3. Statistics

The SPSS statistics software (Version 25, IBM Corp) was used for statistical analysis. The measurement data were first tested for normality and homogeneity of variance, and those with a normal distribution were expressed as the mean ± standard deviation (SD). Data with a non-normal distribution were expressed as a median (lower quartile, upper quartile) [M (Q_L_, Q_U_)]. An independent samples *t*-test or non-parametric test was used to compare two means. A one-way analysis of variance (ANOVA) was used to compare normally distributed data between multiple groups, and pairwise comparisons were further performed if statistical differences were detected. The Least-Significant Difference (LSD) method was used if the variance between groups was equal, and Tamhane’s T2 method was used if the variance was not equal. A non-parametric test (Kruskal–Wallis *H*-test) was used to compare the non-normally distributed data between multiple groups. If statistical differences were found, the Mann–Whitney *U*-test was further performed for pairwise comparisons, and the Bonferroni’s method was used to correct the test level. Categorical variables are given as percentages. The chi-square test was used for comparisons between categorical variables. Correlations were analyzed using Pearson correlation analysis (normally distributed data) or Spearman correlation analysis (non-normally distributed data). The significance level was set at α = 0.05 (two-tailed).

## 3. Results

### 3.1. Baseline participant characteristics

We enrolled 117 patients with BD and 47 healthy volunteers. The groups were not significantly different in terms of age, gender, education level, and smoking status (*P* > 0.05). Among the 117 patients, 110 (94.0%) used atypical antipsychotics, 115 (98.3%) used mood stabilizers, 13 (11.1%) used antidepressants, and 72 (61.5%) used benzodiazepines. The participants with BD had decreased OS (*P* = 0.004) and OI (*P* = 0.005) compared with controls, indicating that they had defects in olfactory function (see Table 1).

**TABLE 1:** Sociodemographic and clinical characteristics of the BD and control groups.

	BD (*n* = 117)	Control (*n* = 47)	χ*^2^/Z*	*P*-value
**Sociodemographics**				
Gender, *N* (%)				
Male	60 (51.3)	25 (53.2)	0.049	0.825
Female	57 (48.7)	22 (46.8)		
Age (years), M (P_25,_ P_75_)	33 (26, 41)	35 (24, 41)	−0.247	0.805
Smoking history, *N* (%)	30 (25.6)	6 (12.8)	3.244	0.072
Education (years), M (P_25,_ P_75_)	11 (9, 13)	11 (9, 12)	−0.519	0.604
**Clinical characteristics**				
Course of disease (years)	8 (4, 13.5)	–		
Psychotic symptoms, *N* (%)	16 (13.7)	–		
HAMD, M (P_25,_ P_75_)	4 (2, 14)	0 (0, 1)		
YMRS, M (P_25,_ P_75_)	3 (0, 14)	0 (0, 0)		
CGI-SI, M (P_25,_ P_75_)	4 (4, 5)	–		
GAF, M (P_25,_ P_75_)	47 (43, 55)	90 (90, 90)		
**Medications**				
Atypical antipsychotics, *N* (%)	110 (94.0)	–		
Olanzapine	45 (38.5)			
Risperidone	16 (13.7)			
Aripiprazole	14 (12.0)			
Quetiapine	19 (16.2)			
Mood stabilizers, *N* (%)	115 (98.3)	–		
Valproate	58 (49.6)			
Lithium	70 (59.8)			
Oxcarbazepine	16 (13.7)			
Antidepressants, *N* (%)	13 (11.1)	–		
Benzodiazepines, *N* (%)	72 (61.5)	–		
Lorazepam	32 (27.4)			
Oxazepam	21 (17.9)			
**Olfactory function**				
OS, M (P_25,_ P_75_)	8 (6, 10.5)	9.3 (7.5, 11.5)	−2.877	0.004*
OI, M (P_25,_ P_75_)	12 (11, 13)	13 (12, 14)	−2.803	0.005*

BD, bipolar disorder; HAMD, Hamilton depression rating scale; YMRS, Young mania rating scale; CGI-SI, clinical general impression scale-severity of illness; GAF, global assessment function; OS, olfactory sensitivity; OI, olfactory identification.

*Value is statistically significant.

### 3.2. Comparison of olfactory function between patients with BD I and BD II

We divided the 117 BD patients into a BD I group (*n* = 86) and a BD II group (*n* = 31). We found no significant differences in age, gender, education level, and smoking status among the BD I, BD II groups and controls, and no significant differences in the course of disease between the BD I and BD II groups (*P* > 0.05). There were more patients with psychotic symptoms in the BD I group and more patients using antidepressants in the BD II group (see Table 2).

**TABLE 2:** Sociodemographic and clinical characteristics of the BD I, BD II, and control groups.

	BD I (*n* = 86)	BD II (*n* = 31)	Control (*n* = 47)	χ*^2^/Z*	*P*-value
**Sociodemographics**					
Gender, *N* (%)					
Male	45 (52.3)	15 (48.4)	25 (53.2)	0.191	0.909
Female	41 (47.7)	16 (51.6)	22 (46.8)		
Age (years), M (P_25,_ P_75_)	33.5 (26, 42.3)	31 (25, 38)	35 (24, 41)	0.557	0.757
Smoking history, *N* (%)	22 (25.6)	8 (25.8)	6 (12.8)	3.245	0.197
Education (years), M (P_25,_ P_75_)	10 (9, 12.3)	12 (9, 15)	11 (9, 12)	0.968	0.616
**Clinical characteristics**					
Course of disease (years)	7.5 (3, 13)	9 (4.5, 15)	–	1.128	0.259
Psychotic symptoms, *N* (%)	24 (27.9)	2 (6.5)	–	6.069	0.014*
HAMD, M (P_25,_ P_75_)	2.5 (1, 11)	14 (4, 23)	–	−4.286	0.000*
YMRS, M (P_25,_ P_75_)	8.5 (1.8, 18)	0 (0, 1)	–	−5.623	0.000*
CGI-SI, M (P_25,_ P_75_)	4 (4, 5)	4 (3, 5)	–	−1.517	0.129
GAF, M (P_25,_ P_75_)	47 (43, 55)	48 (42, 69)	–	−0.581	0.561
**Medications**					
Atypical antipsychotics, *N* (%)	81 (94.2)	29 (93.6)	–	0.016	1.000
Olanzapine	36 (41.9)	9 (29.0)	–	1.584	0.208
Risperidone	14 (16.3)	2 (6.5)	–	1.125	0.289
Aripiprazole	10 (11.6)	4 (12.9)	–	0.000	1.000
Quetiapine	12 (14.0)	7 (22.6)	–	1.247	0.264
Mood stabilizers, *N* (%)	85 (98.8)	30 (96.8)	–	0.557	0.461
Valproate	41 (47.7)	17 (54.8)	–	0.468	0.494
Lithium	55 (64.0)	15 (48.4)	–	2.297	0.130
Oxcarbazepine	11 (12.8)	5 (16.1)	–	0.025	0.874
Antidepressants, *N* (%)	5 (5.8)	8 (25.8)	–	9.222	0.005*
Benzodiazepines, *N* (%)	54 (62.8)	18 (58.1)	–	0.215	0.643
Lorazepam	28 (32.6)	4 (12.9)	–	4.430	0.035*
Oxazepam	14 (16.3)	7 (22.6)	–	0.614	0.433
**Olfactory function**					
OS, M (P_25,_ P_75_)	7.5 (5.5, 9.6)	8.5 (7.5, 12.5)	9.3 (7.5, 11.5)	15.920	0.000*
OI, M (P_25,_ P_75_)	12 (11, 13)	13 (11, 14)	13 (12, 14)	12.185	0.002*

BD, bipolar disorder; BD I, bipolar I disorder; BD II, bipolar II disorder; HAMD, Hamilton depression rating scale; YMRS, Young mania rating scale; CGI-SI, clinical general impression scale-severity of illness; GAF, global assessment function; OS, olfactory sensitivity; OI, olfactory identification.

*Value is statistically significant.

**FIGURE 1:**
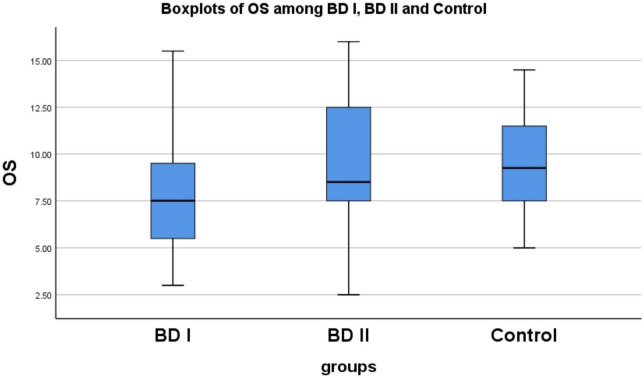
There are statistically significant differences in OS between BD I group and control (*p* < 0.001), similarly between BD I group and BD II group (*P* = 0.008). OS, olfactory sensitivity; BD, bipolar disorder; BD I, bipolar I disorder; BD II, bipolar II disorder.

We found significant differences in OS (χ^2^ = 15.920, *P* < 0.001) and OI (χ^2^ = 12.185, *P* = 0.002) among the BD I, BD II groups and controls. We used the Mann–Whitney *U*-test for pairwise comparison of the OS and OI scores, and the Bonferroni’s method to correct the test level. The adjusted α was 0.017. We found statistically significant differences in OS (*P* < 0.001) and OI (*P* = 0.001) between the BD I group and controls, and in OS (*P* = 0.008) between the BD I and BD II groups (see Supplementary material 1 and Figure 1). These results indicate that impairments in olfactory function may differ according to different subtypes of BD.

### 3.3. Comparison of olfactory function among BD patients according to episode type

We divided the 117 BD patients into Depressive BD (D-BD, *n* = 36), Manic BD (M-BD, *n* = 44), and Euthymic BD (E-BD, *n* = 37) groups according to episode type. We found no significant differences in age, gender, education level, and smoking status among the D-BD, M-BD, E-BD groups and controls, and no significant differences in the course of disease among the D-BD, M-BD, and E-BD groups (*P* > 0.05) (see Table 3).

**TABLE 3:** Sociodemographic data, medications and olfactory function among the D-BD, M-BD, E-BD, and control groups.

	D-BD (*n* = 36)	M-BD (*n* = 44)	E-BD (*n* = 37)	Control (*n* = 47)	χ*^2^/F*	*P*-value
**Sociodemographics**						
Gender (M/F)	21/15	21/23	18/19	25/22	1.091	0.779
Smoking history, *N* (%)	10 (27.8)	12 (27.3)	8 (21.6)	6 (12.8)	3.757	0.289
Age (years)	32.2 ± 8.9	34.9 ± 10.5	35.2 ± 10.5	33.5 ± 10.6	0.695	0.556
Education (years)	12 (9, 14)	10.5 (8, 12)	9 (9, 12)	11 (9, 12)	2.674	0.445
Course of disease (years)	7 (5, 12.3)	11 (1.3, 15)	6 (3, 12.5)	–	1.338	0.512
**Medications, *N* (%)**						
Atypical antipsychotics	32 (88.9)	41 (93.2)	37 (100.0)	–	4.092	0.129
Olanzapine	8 (22.2)	18 (40.9)	19 (51.4)	–	6.720	0.035*
Risperidone	7 (19.4)	7 (15.9)	2 (5.4)	–	3.344	0.188
Aripiprazole	1 (2.8)	8 (18.2)	5 (13.5)	–	4.583	0.101
Quetiapine	9 (25.0)	6 (13.6)	4 (10.8)	–	3.052	0.217
Mood stabilizers	36 (100.0)	43 (97.7)	36 (97.3)	–	0.926	0.629
Valproate	24 (66.7)	18 (40.9)	16 (43.2)	–	6.122	0.047*
Lithium	15 (41.7)	28 (63.6)	27 (73.0)	–	7.866	0.020*
Oxcarbazepine	6 (16.7)	4 (9.1)	6 (16.2)	–	1.259	0.533
Antidepressants	12 (33.3)	0 (0)	1 (2.7)	–	26.149	0.000*
Benzodiazepines	29 (80.6)	26 (59.1)	17 (45.9)	–	9.413	0.009*
Lorazepam	13 (36.1)	15 (34.1)	4 (10.8)	–	7.491	0.024*
Oxazepam	8 (22.2)	6 (13.6)	7 (18.9)	–	1.026	0.599
**Olfactory function**						
OS	8.4 ± 3.0	7.3 ± 2.6	9.4 ± 3.3	9.7 ± 2.6	6.695	0.000*
OI	12.5 (10.3, 14)	12.5 (11, 13)	12 (11, 13.5)	13 (12, 14)	7.927	0.048*

BD, bipolar disorder; D-BD, depressive bipolar disorder; M-BD, manic bipolar disorder; E-BD, euthymic bipolar disorder; M/F, male/female; OS, olfactory sensitivity; OI, olfactory identification.

*Value is statistically significant.

**FIGURE 2:**
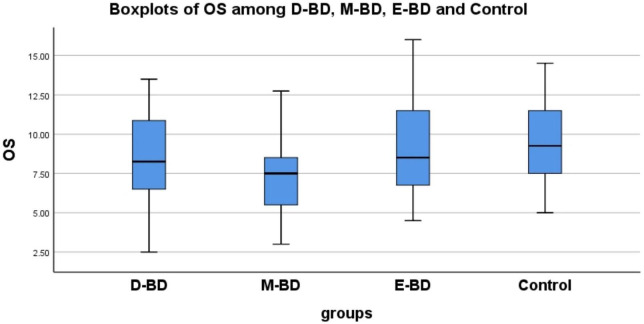
There are statistically significant differences in OS between D-BD, M-BD, and control (*P* = 0.036, *P* < 0.001) and between M-BD and E-BD (*P* = 0.001). OS, olfactory sensitivity; BD, bipolar disorder; D-BD, depressive bipolar disorder; M-BD, manic bipolar disorder; E-BD, euthymic bipolar disorder.

Because the OS of the different subgroups was normally distributed and the OI was non-normally distributed, we used different statistical methods to process these datasets. We found significant differences in OS (*F* = 6.695, *P* < 0.001) and OI (*F* = 7.927, *P* = 0.048) among the D-BD, M-BD, E-BD groups and controls, respectively. Further LSD tests with multiple comparisons showed statistically significant differences in OS between the D-BD, M-BD groups and controls (*P* = 0.036, *P* < 0.001) and between the M-BD and E-BD groups (*P* = 0.001). We used the Mann–Whitney *U*-test to conduct pairwise comparisons of OI scores, and the Bonferroni’s method to correct the test level. The adjusted α was 0.0083. We found no statistically significant differences in OI scores among the groups. Nonetheless, our results showed that defects in olfactory function differ among BD patients according to episode type (see Supplementary material 2 and Figure 2).

### 3.4. Correlation between OS or OI and clinical characteristics in BD patients

We used Pearson correlation analysis or Spearman’s correlation to examine the relationship between OS or OI and clinical characteristics in the BD I and BD II groups, respectively. The results revealed that OI was positively correlated with the YMRS score in patients with BD I (*r* = 0.235, *P* = 0.030) while OS was negatively correlated with the HAMD score in patients with BD II (*r* = −0.428, *P* = 0.016) (see Table 4). Thus, greater mania severity in BD I patients was associated with better olfactory identification, while more severe depressive symptoms in BD II patients were associated with lower olfactory sensitivity.

**TABLE 4:** Correlation between OS or OI and clinical characteristics in BD I and BD II patients (α = 0.05).

Variable	HAMD		YMRS		CGI-SI		GAF	
	*r*	*P*	*r*	*P*	*r*	*P*	*r*	*P*
BD I: OS	−0.110	0.311	−0.083	0.447	−0.027	0.803	0.060	0.580
OI	0.011	0.917	0.235	0.03*	0.077	0.483	−0.161	0.139
BD II: OS	−0.428	0.016*	−0.214	0.248	−0.290	0.113	0.114	0.542
OI	−0.063	0.736	0.140	0.453	−0.043	0.817	0.022	0.906

OS, olfactory sensitivity; OI, olfactory identification; BD, bipolar disorder; BD I, bipolar I disorder; BD II, bipolar II disorder; HAMD, Hamilton depression rating scale; YMRS, Young mania rating scale; CGI-SI, clinical general impression scale-severity of illness; GAF, global assessment function.

*Value is statistically significant.

We similarly analyzed the correlations between OS or OI and clinical characteristics in all BD patients according to episode type. However, we found no significant associations between OS or OI and any of the symptom or social function scales.

## 4. Discussion

The relationship between olfaction and mental illness is receiving increasing attention from researchers. As brain regions related to olfaction partially overlap with those related to mental diseases (36), the integrity of these areas can be investigated by studying olfactory function with non-invasive and effective methods. However, further investigation using neuroimaging is needed to elucidate the neural correlates of olfactory function in these disorders (37). Brain regions involved in olfaction, such as the prefrontal cortical areas, striatum, and amygdala, are often implicated in the physiopathology of BD. This could explain some of olfactory abnormalities described in BD patients. Furthermore, some studies about olfaction have revealed that OI defects could act as potential markers for BD. However, whether olfactory deficits are a state or trait marker of BD is still controversial (38, 39).

### 4.1. Comparison of olfactory function between BD patients and healthy controls

Our data indicated that all 117 patients with BD had decreased OS and OI performance compared with the healthy controls. Thus, BD patients may have impaired olfactory function that manifests as a decline in OS and OI ability. Accordingly, olfactory dysfunction may be a specific biological biomarker that could facilitate the early diagnosis or treatment of BD. Although some previous studies supported our results (21, 22, 27), this was not the case for others (23–25, 28–30). This could be explained by differences in sample size and the types of episodes experienced by the enrolled BD patients. For instance, the sample size of most previous studies was relatively small. Regarding the composition of BD patients in different studies, some only had depressive episodes (23), some were only in the euthymic state (29), and some were in a stable state (24). However, some studies mixed patients with BD and depression (30).

Chen et al. (16) found OI and OS deficits in patients with generalized anxiety disorder, compared with healthy controls. And a few studies in other anxiety disorders such as post-traumatic stress disorder (17, 18) and obsessive-compulsive disorder (19, 20) reported the deficits of olfactory function. Given the high prevalence of anxiety disorders (up to 50%) among BD patients (40), this comorbidity might aggravate the impairment of olfactory function. However, previous olfaction studies in panic disorder have yielded conflicting results, which showed higher OS of these patients as well as a greater olfactory awareness (41) or normal OI (42) compared to the healthy controls. Psychotic symptoms are similarly common in BD patients. More than half experience psychotic features in their lifetime (43). van Bergen et al. reported in 2019 that the vast majority (73.8%) of BDI patients had experienced psychotic symptoms in their lifetime (44). Psychotic symptom comorbidities may contribute to the occurrence of impaired olfactory function in BD patients (26).

Whether or not olfactory dysfunction can be used as a biological marker of BD is of great importance for the early diagnosis of BD. Unfortunately, research on this topic is sparse and inconsistent. Only a few studies have reported results suggesting that impairment of OI may be a potential trait marker for BD (21, 22, 26, 27). Cumming et al. (21) examined OI on the UPSIT accuracy between BD and schizophrenia patients and showed that OI deficit was present in both disorders compared with healthy participants, but BD patients were less severely affected than schizophrenic patients. Lahera et al. reported that OI deficits persisted in BD patients undergoing remission (22). Li et al. (27) suggested OI impairment is a trait, but not a disease-specific marker in BD, by comparing olfactory function between all BD patients in different mood episodes and controls, as well as between BD, major depressive disorder, schizophrenia and controls.

These findings strongly suggest that OI impairment may be a potential marker for BD; however, further study is needed before use in the clinical setting. Given that OI deficits are also seen in other psychiatric disorders, it has relatively low specificity as a potential diagnostic tool for BD. Further studies should be carried out to develop diagnostic tools with high sensitivity and specificity for olfactory function, improve existing olfactory indicators, and even combine them with other biological indicators. Advances stemming from these works should help improve the sensitivity and specificity of early diagnostic tools for BD, promote the standardization of treatment, and improve prognosis in patients.

### 4.2. Comparison of olfactory function between patients with BD I and BD II

Few studies have examined olfactory function in BD patients according to subtypes. The results of this study showed decreased OS and OI in BD I patients compared with controls, which was partially consistent with previous studies. In 2018, Kamath et al. examined OI and OS in 42 BD I patients, 45 BD II patients, and 71 controls. They found that the OI in BD I patients was lower than that of the controls, while the OS in 18 BD I patients and 22 BD II patients was not significantly different from that in the controls (26). Possible explanations for this inconsistency with respect to the present study are the sample size and methods used to measure olfactory function.

In this study, we found no significant differences in olfactory function between BD II group and the controls. This is similar to the results of Kamath (26), but contrary to the results reported by Lovdahl et al. (45). Lovdahl et al. used the symptom list questionnaire to assess olfactory impairment in patients with BD II. They found that 14% of patients with BD II (*n* = 21) had olfactory impairment, while the olfactory impairment rate of the controls (*n* = 21) was 0%. However, they performed semi-structured interviews, and thus used a subjective method to evaluate olfactory function, rather than an olfactory assessment of OI or OS *via* the UPSIT or SST.

According to the DSM-5, the key to distinguishing BD I from BD II is the presence of manic episodes in BD I and hypomanic episodes in BD II. The conditions also differ in terms of lifetime prevalence (46, 47), age of onset (48), clinical manifestations (49), comorbidity pattern (50), and the degree of impairment with respect to psychosocial function (51). A study from 2022 examined the genetic overlap and distinction between BD I and BD II *via* integrative post-GWAS analyses, and found genetic differences with a set of candidate genes distinguishing BD I from BD II (52). Similarly, Liu et al. used resting state functional magnetic resonance imaging (fMRI) and data preprocessing technology to uncover shared and unique neurobiological mechanisms between BD I and BD II (53). They reported that the dynamic amplitudes of low-frequency fluctuation (dALFF) values in BD II patients were significantly higher than those in BD I patients in the right superior temporal gyrus, which indicated that activity in this region could act as a potential biomarker for the differential diagnosis of BD subtypes. Kamath et al. (26) examined olfactory function (including OI, OS, odor discrimination, and odor hedonic processing performance) in patients with BD I, BD II, major depressive disorder, anxiety, and controls, and compared olfactory scores between diagnostic groups and controls. However, they did not conduct pairwise comparisons between diagnostic groups (i.e., BD I vs. BD II). To the best of our knowledge, the current study is the first to examine olfactory function *via* pairwise comparisons between BD I and BD II patients. In our study, the OS in BD I patients was significantly decreased compared with that in BD II patients. This result is consistent with the views of Hardy et al. (24), who believed that diminished OS could predict social impairment, and Judd et al. (51), who concluded that BD I patients had poorer psychosocial function than BD II patients.

We found that BD I patients had OS impairment compared with BD II patients and controls, suggesting that among BD patients reduced OS may be prominent only in those diagnosed with BD I. Our findings provide evidence for BD I as an independent subtype and suggest that OS can be a potential biomarker for distinguishing BD I from BD II. Considering that the guidance-based medication principles (especially the use of antidepressants) and prognosis of BD I and BD II differ, the differentiation of BD I and BD II is of great clinical significance, and may advance appropriate treatment approaches for these patients.

### 4.3. Comparison of olfactory function in BD patients according to episode type

We found decreased OS in BD patients with manic and depressive episodes compared with controls, and decreased OS in patients with manic episodes compared with euthymic patients. These results are in accordance with Li et al. (27), who reported that only depressive and manic BD patients (but not euthymic BD patients) had poor OS compared with control subjects. However, our findings are inconsistent with the findings of Kazour et al. (28) and Swiecicki et al. (23), who found no significant differences in OS score between depressive BD patients and controls.

We found no statistically significant differences in OI scores among BD groups according to episode type, which is similar to the findings reported by Swiecicki et al. (23) and Kazour et al. (28). However, this is contrary to Li et al. (27), who demonstrated OI deficits in all BD patients (depressed, manic, and euthymic subgroups), indicating that OI may be a trait marker for BD. These inconsistencies are likely caused by the heterogeneity of the patient groups (different sociodemographic and clinical characteristics) as well as different sample sizes.

Compared with controls, we found no significant differences in OS or OI in BD patients in the euthymic stage. This result is in line with the views of Negoias et al. (29). However, Lahera et al. reported that BD patients showed a significant deficit in OI (measured *via* UPSIT) compared with healthy controls (22). This inconsistency with respect to the present study may be explained by the different patient clinical characteristics and the methods of measuring olfactory function. In the study of Lahera et al., the patients were older, the course of disease was longer, and the proportion of smoking was higher. These factors may have had adverse effects on olfactory function. At present, smoking is generally considered to be negatively associated with olfactory ability in a dose-related manner in smokers (54–56). In addition, Krüger et al. reported that euthymic BD patients with event-triggered episodes had increased OS compared with those without such episodes, although their study had no healthy control group and the sample size was very small (31).

Our data indicate that BD patients who experience manic or depressive episodes might have deficits in olfactory function, mainly related to OS. In contrast, the OS and OI in euthymic patients did not significantly differ from those in controls. Our data indicate that OS may be a promising biomarker of BD state that could be used to differentiate the acute phase of BD from the euthymic state. This could be helpful when monitoring the therapeutic effects of medication. But in contrast to longitudinal studies, this cross-sectional comparison of olfactory function between the acute phase (mania or depression) and euthymic state of BD has limitations and increased bias. A more robust evaluation would have been a prospective comparison of the same patients in the depressive or manic phase and after remission. Further prospective and longitudinal follow-up studies are needed to explore the dynamic changes in olfactory function according to medication use in BD patients.

### 4.4. Correlation between OS or OI and clinical characteristics in BD patients

In this study, we also explored how olfactory function related to emotional symptoms and social function. Our results showed that OS in BD II patients was negatively correlated with depressive symptoms. That is, more severe depressive symptoms were related to a lower OS. This is similar to the views of Li et al., who found that OS was negatively correlated with HAMD scores (27). This is also consistent with the classic symptoms of depressive syndrome (including depressed mood, loss of interest or pleasure, slow thinking, decreased activity, decreased appetite, hypoesthesia, suicidal ideation, or even suicide attempts). For instance, Parker et al. observed that almost all participants with BD reported smells as weaker (e.g., dull, degraded, less strong) when depressed (57). However, this is inconsistent with the view of Hardy et al., who reported that depressive symptoms were related to increased OS (24). The reason for this inconsistent result may be the heterogeneity of the research methods (all participants were clinically stable) or the comparatively small study sample sizes (20 patients) in the study of Hardy et al.

Our data also indicated that OI in BD I patients was positively correlated with manic symptoms, that is, the more severe the manic symptoms in BD patients, the better the OI ability. This is to some extent consistent with the classic symptoms of hypomanic or manic syndrome (including elevated mood and increased energy, attention, or goal-directed activity). Parker et al. reported that almost all participants with BD described enhanced olfaction, using terms like “sharper,” “clearer,” “stronger,” and “more intense” during hypomanic/manic states (57, 58). Indeed, the DSM-5 states that during manic episodes, some BD patients may feel a “sharper sense of smell” (59). This is inconsistent with the views of Hardy et al. (24) and Li et al. (27), who both reported that smell identification was unrelated to the clinical features of BD.

Our findings showed significant associations between olfactory function and emotional symptoms and confirmed the correlation between olfaction and emotion. The common brain regions shared by BD and olfaction might account for some of the olfactory alterations described in BD patients. Currently, there are only a few studies on the relationship between BD and olfactory impairment, and the findings have been inconsistent. Further study combined with new imaging techniques should provide new evidences for clarifying the pathophysiological mechanisms of BD.

Previous studies have shown that olfactory function in patients with BD is related to psychosocial cognition and social function. For instance, Hardy et al. found that BD patients with higher OS performance had lower levels of social fear and social avoidance, along with higher independence and better employment status (24). Furthermore, Cumming et al. found a positive correlation between OI and social competence in patients with BD (21). Although we also compared the relationship between olfactory function and social function in the present study, no statistically significant results were obtained. It may be that the GAF scale utilized to evaluate social function in this study was subjective and not sufficiently sensitive.

We did not find a correlation between OS or OI and the clinical characteristics of BD according to BD diagnosis or different episode types, respectively. That is, there were no significant associations between OS or OI and any of the symptom or social function scales. As previous studies have produced mixed results, further studies are needed.

## 5. Conclusion

Our study demonstrated that BD patients have impaired olfactory function, and that impairments in OS vary between patients with BD I and BD II. To the best of our knowledge, this study is the first to compare olfactory function *via* pairwise comparisons in BD I and BD II patients. Our data provide evidence for the differential diagnosis of BD I and BD II and suggest that OS may have potential as a biomarker for the different subtypes of BD. Furthermore, we revealed that BD patients exhibit decreased OS during manic and depressive episodes, while OS in euthymic BD patients is similar to that in healthy controls. This indicates that OS performance may function as a state-related biomarker that could be used to differentiate the acute phase from the euthymic state of BD.

This study has several limitations. First, this study used a cross-sectional design, which has limitations and increased bias compared with longitudinal studies. To reduce this bias, we matched all groups (including controls) according to age, gender, education level, and smoking status. Further prospective research is necessary to explore the dynamic changes in olfactory function. Second, the olfactory detection method used in this study is comparatively subjective, which could affect the results. In future work, it will be necessary to apply objective olfactory function detection methods, such as olfactory event-related potentials, to minimize the subjective error. Furthermore, we failed to exclude the influence of patient medication regimen on olfactory function, which could include many confounding factors such as the type, dose, and treatment schedule of psychotropic drugs, electric shock treatment, transcranial magnetic stimulation treatment. Subsequently, a prospective and more rigorous control study should be carried out to explore the influence of psychotropic drugs and physical therapy on olfactory function in BD patients. Finally, our study did not combine with the latest imaging technology to reveal the neural pathways associated with olfactory function in BD patients. Future studies should illuminate the neuroimaging correlates of olfactory function in BD patients with different subtypes and episodes.

## Data availability statement

The original contributions presented in this study are included in the article/Supplementary material, further inquiries can be directed to the corresponding authors.

## Ethics statement

The studies involving human participants were reviewed and approved by the Ethics Committee of Shunde Wu Zhongpei Hospital, Foshan. The patients/participants provided their written informed consent to participate in this study. Written informed consent was obtained from the individual(s) for the publication of any potentially identifiable images or data included in this article.

## Author contributions

CL: concept and design, data analysis and interpretation, statistical analysis, and drafting the manuscript. LH: data acquisition, analysis and interpretation, and statistical analysis. LZ: design and critical revision of the manuscript. YZ, JY, and FW: data acquisition and analysis. CC: data supervision and critical revision of the manuscript. All authors read and approved the final manuscript.
