# Supplementary material for: Variations in olfactory function among bipolar disorder patients with different episodes and subtypes
Source: Front Psychiatry. 2023 Mar 20;14:1080622. doi: 10.3389/fpsyt.2023.1080622 (PMC10067908; doi:10.3389/fpsyt.2023.1080622)
Supplement: Supplementary file 1 [file Table_1.pdf]

Supplementary Material 1

Pairwise comparison of OS and OI between groups (Mann–Whitney *U*-test, Bonferroni correction)

Pairwise group	Z	P Value	α’
OS: BD I vs BD II	-2.633	0.008*	0.017
BD I vs Control	-3.681	0.000*	0.017
BD II vs Control	-0.092	0.927	0.017
OI: BD I vs BD II	-1.932	0.053	0.017
BD I vs Control	-3.419	0.001*	0.017
BD II vs Control	-0.473	0.636	0.017

OS: olfactory sensitivity; OI: olfactory identification; BD: bipolar disorder; BD I: Bipolar I disorder; BD II: Bipolar II disorder.

*statistically significant.

α (level of test) = 0.05; *α*’ (adjusted level of test) = 0.05/3 (0.017).
