# Supplementary material for: Variations in olfactory function among bipolar disorder patients with different episodes and subtypes
Source: Front Psychiatry. 2023 Mar 20;14:1080622. doi: 10.3389/fpsyt.2023.1080622 (PMC10067908; doi:10.3389/fpsyt.2023.1080622)
Supplement: Supplementary file 2 [file Table_2.pdf]

Supplementary Material 2

Pairwise comparison of OS and OI between groups (LSD or Mann–Whitney *U*-test, Bonferroni correction)

Pairwise group	SE/Z	P Value	α'
OS: D-BD vs M-BD	0.636	0.077	----
D-BD vs E-BD	0.663	0.132	----
D-BD vs Control	0.627	0.036*	----
M-BD vs E-BD	0.632	0.001*	----
M-BD vs Control	0.594	0.000*	----
E-BD vs Control	0.622	0.607	----
OI: D-BD vs M-BD	-0.128	0.898	0.0083
D-BD vs E-BD	-0.016	0.951	0.0083
D-BD vs Control	-2.007	0.045	0.0083
M-BD vs E-BD	-0.304	0.761	0.0083
M-BD vs Control	-2.287	0.022	0.0083
E-BD vs Control	-2.413	0.016	0.0083

OS: olfactory sensitivity; OI: olfactory identification; LSD: Least-Significant Difference; SE: standard error; BD: bipolar disorder; D-BD: depressive bipolar disorder; M-BD: manic bipolar disorder; E-BD: euthymic bipolar disorder.

*statistically significant.

α (level of test) = 0.05; α' (adjusted level of test) = 0.05/6 (0.0083).
